# Fracture Resistance in Non-Vital Teeth: Absence of Interproximal Ferrule and Influence of Preparation Depth in CAD/CAM Endocrown Overlays—An In Vitro Study

**DOI:** 10.3390/ma15020436

**Published:** 2022-01-07

**Authors:** Lucía Barallat, María Arregui, Sandra Fernandez-Villar, Blanca Paniagua, Andrés Pascual-La Rocca

**Affiliations:** 1 Department of Periodontics, Faculty of Dentistry, Universitat Internacional de Catalunya, 08195 Sant Cugat del Valles, Spain; lbarallat@uic.es (L.B.); bpaniagua@uic.es (B.P.); ampascual@uic.es (A.P.-L.R.); 2 Department of Dentistry, Faculty of Dentistry, Universitat Internacional de Catalunya, 08195 Sant Cugat del Valles, Spain; 3 Department of Restorative Dentistry, Faculty of Dentistry, Universitat Internacional de Catalunya, 08195 Sant Cugat del Valles, Spain; sandrafernandez@uic.es

**Keywords:** fracture resistance, overlay restoration, endocrown, ferrule, premolars

## Abstract

There is ample evidence to support the use of endocrowns to restore endodontic teeth. However, the influence of the position of the interproximal margins on fracture strength has not yet been studied. The aim was to determine the relationship between the apicocoronal position of the interproximal restorative margins and fracture resistance in nonvital teeth restored with CAD/CAM endocrown overlays. Forty extracted human maxillary premolars were prepared for endocrown overlay restorations without ferrule on the interproximal aspects and classified according to the position of the interproximal restoration margins in relation to the alveolar crest: 2 mm (group A), 1 mm (group B), 0.5 mm (group C), and 0 mm (group D). Fracture strength was measured using a universal testing machine applying a compressive force to the longitudinal tooth axis. Group A had a mean fracture resistance of 859.61 (±267.951) N, group B 1053.9 (±333.985) N, group C 1124.6 (±291.172) N, and group D 780.67 (±183.269) N, with statistical differences between groups. Group C had the highest values for fracture strength compared to the other groups (*p* < 0.05). The location of the interproximal margins appears to influence the fracture resistance of CAD/CAM endocrown overlays. A distance of 0.5 mm between the interproximal margin and the alveolar crest was associated with increased fracture resistance.

## 1. Introduction

It is currently accepted that restorative treatments should be associated with an aesthetic result that meets the characteristics of a natural dentition. In order to preserve the maximum dental structure, as well as to limit the risk of fracture, the minimally invasive approach with a minimum biological compromise is one of the standards of restorative dentistry [1,2].

Traditionally, the treatment of endodontically treated teeth with extensive loss of tooth structure included full-coverage crowns supported by posts and cores, and a sufficient ferrule was considered to be critical to ensure their long-term survival [3,4,5]. Morgano & Brackett [6] defined ferrule as “incorporating a crown with a 360° collar that surrounds the perimeter of the prepared parallel dentine walls and extending cervical to the shoulder of the tooth preparation” which provides a protective effect by reducing the stresses within a tooth [7]. Several studies have shown that at least 1 mm of ferrule height significantly improved fracture resistance when restoring endodontically treated teeth [7,8,9,10].

Also, when dealing with a tooth-supported restoration, biologic width has to be considered. This concept was first introduced by Gargiulo in 1961 [11] as the composition of junctional epithelium and connective tissue fibers attaching and surrounding the tooth in order to protect periodontal ligament and the alveolar bone and measures around 3 mm including the sulcular depth; these results where later confirmed by Vacek [12]. It has been observed that respecting these dimensions when restoring teeth is necessary to preserve healthy soft tissues. On the contrary, if a prosthetic margin invades these supracrestal attached tissues, an inflammatory response will take place resulting in increased probing pocket depth, gingival recession and/or periodontal attachment loss [13,14,15]. In cases with advanced tooth destruction due to caries or trauma, treatments like orthodontic extrusion or periodontal crown lengthening have been proposed to obtain healthy supracrestal dental structure, in order to avoid biologic width invasion and gain ferrule. Crown lengthening has been defined as a surgical procedure that involves the removal of soft and hard tissues in order to gain retention and resistance of tooth structure above the alveolar crest level [16,17]. When using full-coverage crowns, a minimum of 4 to 5 mm tooth height between the crown margin and the alveolar crest has been proposed in order to have sufficient ferrule and avoid biologic width invasion [18].

The field of restorative materials has undergone a major shift in recent years [19,20,21,22]. The improvements in the adhesive techniques have allowed the use of partial restorations, preserving maximum dental structure with a minimally invasive approach [23]. They have been presented as an alternative treatment to full-coverage crowns supported by posts and cores in teeth with advanced destruction [24]. According to the number of affected tooth cusps, restorations can be classified as inlays (without covering the cusps), onlays (covering at least 1 cusp), and overlays (covering all cusps) [25]. In this context, also the “endocrown” concept has been proposed, which consists of a single block overlay, which penetrates inside the pulp chamber, with the aim of improving the adhesive retention of the restoration [26,27,28]. The design of the preparation may vary according to the descriptions of the authors. One example is the description used by Bindl & Morman in 1999 [29], in which the authors defined that the occlusal part (the cuspal covering) had to present a thickness between 3 and 5 mm while inside the pulp chamber the depth could range from 1 to 4 mm. Other authors similarly described a pulp chamber depth between 2 and 5 mm [28,30,31].

Restoring endodontically treated teeth using post and core restorations with a full coverage crown has traditionally been the treatment of choice, because the full crown provides an optimal cusp protection. The drawback is that this type of rehabilitation requires more tooth structure to be removed, making the tooth weaker, and therefore increasing the risk of fracture [22,32,33]. In comparison, endocrowns reduce the need for extra tooth preparation to obtain a geometrically retentive cavity [23]. Since this type of cavity provides macromechanical retention by the action of the walls inside the pulp chamber, it combines with micromechanical retention by the luting agent to provide adhesive retention [22,33,34].

The advantages of endocrowns, in addition to preserving more tooth structure [22,32,33], are that the interface between the different materials used, root dentine and indirect restoration is reduced [34]; they have better mechanical properties [22,32,33]; high biocompatibility [33]; good esthetics [22,33]; and reduced clinical time [22,33] and treatment costs [22].

The use of endocrowns has increased with the advent of CAD/CAM materials, because they are a monoblock restoration, making them faster than with conventional materials [22]. Currently, there is a wide variety of CAD/CAM materials that can be used to perform endocrowns such as zirconia, lithium disilicate, hybrid ceramics and composite [22,32,34]. Hybrid ceramics and composite blocks versus zirconia and lithium disilicate reduce the post-processing time, as they do not require sintering [32], and they are materials with a modulus of elasticity similar to dentine, which makes them less brittle than ceramics [32,34].

The extension of interproximal caries often leads to the loss of a large amount of dental structure, which occasionally implies the need of endodontic treatment. Usually in these situations, the buccal and lingual walls are kept intact of lesion. For this reason, the analysis of the cavity design is of great importance, especially of the mesial and distal surfaces in order to achieve the maximum resistance of the restoration as well as to preserve dental structure, both occlusally, with a minimum thickness of 2 mm, and inside the pulp chamber.

To our knowledge, there is no sufficient or clear evidence regarding the influence of the preparation design when restoring endodontically treated premolars with CAD/CAM resin composite overlay endocrowns. Therefore, the purpose of this in vitro investigation was to determine if the distance between the interproximal restoration margins and the alveolar bone crest has an influence on fracture resistance.

## 2. Materials and Methods

### 2.1. Groups Distribution and Specimens Preparation

**Figure 1:**
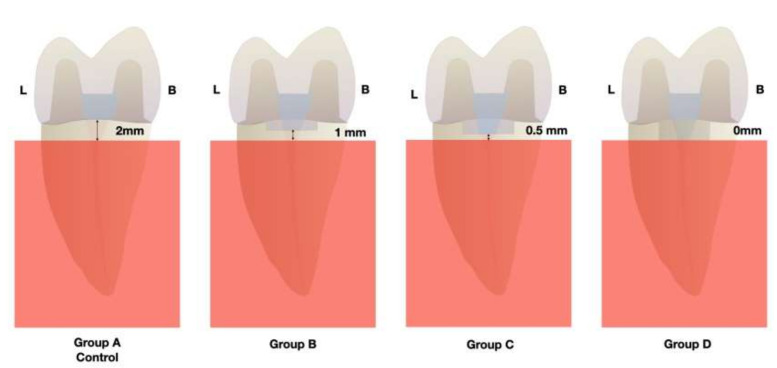
Proximal view of the different locations of the restorative margin in the different treatment groups.

This in vitro study was conducted with the approval of the Research’s Board of the Dental Faculty on February 2013 (PER-ELM-2013-01) in the Universitat Internacional de Catalunya (Barcelona, Spain). Forty healthy human maxillary first premolars with a similar anatomy and no signs of injury or disease were carefully selected. To avoid possible dehydration of the tissues after extraction, all teeth were preserved in distilled water at room temperature. Subsequently, the teeth were mounted individually with the roots embedded in blocks of self-curing acrylic resin (Paladur, Heraeus Kulzer, Hanau, Germany). In all cases, the CEJ was placed 2 mm above the acrylic resin, simulating the anatomic relation with the “alveolar crest”. Subsequently, and randomly, teeth were assigned to four different groups (10 teeth per group) [30,35]. The allocation to the type of treatment of each group was made according to a computer-based block randomization table (Microsoft Office Excel, Microsoft, Redmond, WA, USA) created before the initiation of the study:

- Group A (control group): the restoration margin was placed at the level of the cement-to-enamel junction (CEJ), 2 mm above the simulated alveolar crest.
- Group B: the restoration margin was placed 1 mm under CEJ in mesial and distal walls, 1 mm above the simulated alveolar crest.
- Group C: the restoration margin was placed at 1.5 mm under CEJ in mesial and distal walls, 0.5 mm from above the simulated alveolar crest.
- Group D: the restoration margin was placed at 2 mm under CEJ in mesial and distal walls, at the level of simulated alveolar crest (Figure 1).

All teeth received a full endodontic treatment, and then, following a minimally invasive approach, with a minimum tooth preparation, simulating the extension that caries usually present in the interproximal area, internal and interproximal cavities were prepared. The apical mesial and distal margins were located according to the assigned treatment group at 0 mm, 1 mm, 1.5 mm, and 2 mm under CEJ. The preparation inside the pulp chamber was located at the same level of the interproximal cavities with respect to the bone crest. At the buccal and lingual aspects a marginal preparation was done in order to provide ferrule. Due to the common characteristics of endocrowns and overlays in the chosen design, it could be considered an endocrown overlay (Figure 1).

Next, root canals were sealed and a three-step etch and rinse adhesive system was applied (Adper Scotchbond^®^ Multi-Purpose Plus, 3M ESPE, St. Paul, MN, USA). Afterwards, dentin was covered with a thin resin composite layer (Filtek Z-250^®^, A2, 3M ESPE, St. Paul, MN, USA), and light-cured for 20 s per area (Bluephase, Ivoclar Vivadent, Schaan, Liech-tenstein; 1200 mW/cm^2^). Then, a 2 mm cuspal reduction (all of the remaining walls had more than 1 mm of thickness) with a chamfer margin using cylindrical 80 μm and 40 μm diamond burs (Intensiv S.A, Montagnola, Switzerland) was prepared in the buccal and palatal walls. Lastly, the enamel margins were finished with fine 25μm particle size diamond bur (Intensiv S.A, Montagnola, Switzerland) [36,37]. Subsequently, an optical impression of the cavity was taken using Trios 3^®^ digital intraoral camera system (3 Shape, Copenhagen, Denmark) to obtain a digital model.

Customized endocrown overlays for each included tooth were designed with the same occlusal table using the 3Shape Trios Design software and subsequently milled from nano-ceramic resin CAD/CAM blocks, (A2, HT Lava™ Ultimate CAD/CAM Restorative, 3M ESPE) using the LYRA^®^ milling machine (LYRA^®^, Paris, France).

Before cementation, the restorations were tested and adapted to the cavities and, later on, their internal surfaces were conditioned by airborne-particle abrasion with 30 μm aluminum oxide at 0.25 MPa pressure and silane was applied on the surfaces and air-dried after 60 s. Then, the cavity was abraded with 30 μm aluminum oxide particles. Subsequently, enamel margins were etched using 37.5% orthophosphoric acid for 30 s (Scotch Bond, 3M Espe), dentin for 15 s and then rinsed and dried. A primer agent was applied for 30 s and blow-dried in the surfaces with exposed dentin and a light-curing bonding resin (Adper Scotchbond^®^ Multi-Purpose Plus, 3M ESPE) onto the cavity surface.

The same light-curing bonding resin used on teeth was applied to the restoration but without polymerization. Previously heated (at 50 °C to reduce viscosity), light-curing composite (Filtek Z-250^®^, A2, 3M ESPE) was applied as a luting material into the floor and walls of the cavity [38]. Then, resin restorations were placed and light-cured for 10 s, excess luting composite was removed using a spatula, and a complete 60 s polymerization per surface was performed, and the excess of cement was polished [37,38,39]. After the luting procedure, teeth were stored at room temperature for 24 h, before the fracture resistance test.

### 2.2. Loading Test

**Figure 2:**
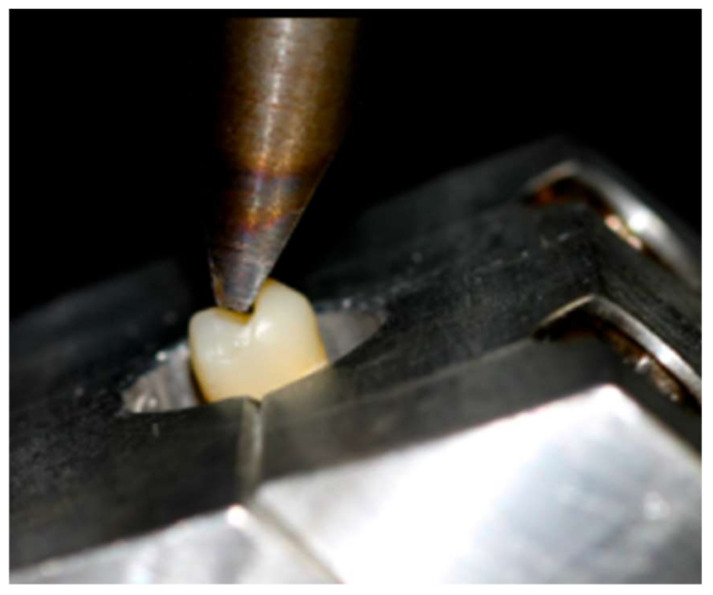
Loading test.

Each specimen was mounted in a custom-made device that ensured the force application at the appropriate angle, which was checked before the compressive load to ensure contact with the internal slope of the palatal cusps. The specimens were located at a 30° angle to the longitudinal axis of the tooth [35,40] in a universal testing machine (Quasar 5, Galdabini, Italy), with a stainless steel attachment on the tip with a dimension of 12.93 mm in length (mesio-distal) and 2.54 mm in width (bucco-palatal), and a cross-head speed of 1 mm/min until fracture. Failure load was determined in Newtons (N) (Figure 2).

### 2.3. Statistical Analysis

Statistical analysis was made with the SPSS 25.0 software package (SPSS Inc., Chicago, IL, USA). The normality of data distribution was tested using Kolmogorov-Smirnov and Shapiro-Wilk tests. Two-way analysis of variance (ANOVA) was used to compare the fracture resistance means among the four groups. Significance level was set at 95% confidence level and 5% beta error. Confidence level of 95% and *p* < 0.05 was considered to be statistically significant.

## 3. Results

Group mean fracture resistance is reported in Table 1. Group A (control group) had a mean fracture resistance of 859.61 N; group B, 1053.90 N; group C, 1124.60 N and group D, 780.67 N being this last group, the one that presented the lowest fracture resistance of all groups. The ANOVA test showed statistically significant differences in fracture strength (*p* = 0.027) among all groups. Group C presented the highest fracture resistance in comparison to the other groups (Figure 1).

## 4. Discussion

The present study was designed to evaluate whether there was a relationship between the fracture resistance of endocrowns with a margin at different depths in the interproximal areas in relation to the alveolar crest, as there is scarce information on this subject, and it was observed that there is a relationship between the location of the cavity margin and the alveolar crest, with a higher resistance to fracture when the distance was 0.5 mm. Advanced loss of dental structure in need of endodontic treatment is a relatively frequent complication found in maxillary premolars [31,41,42]. Different treatment options may range from full preparations, including metal-ceramic crowns, to more conservative approaches such as indirect endocrowns and overlay restorations [43]. Millable resin composite blocks have recently been introduced for use with CAD/CAM systems. In comparison with ceramic materials, these blocks have higher flexural strength and resilience modulus, more similar to the characteristics of dentin [32,44]. When analyzing fracture resistance of teeth restored with indirect resin composite in comparison with ceramic restorations, some in vitro studies have shown no differences between these materials [39] or even a better outcome when a composite restoration is used [31]. Regarding endocrowns, nanocomposite and lithium disilicate glass-ceramic seem to perform better than other materials [45].

Clinically, the maximum posterior occlusal forces range from 400 to 890 N in the molar region and from 222 to 445 N in the premolar area [46]. In the present study, only premolars were included, showing mean fracture loads of 954.69 N, which is above the mentioned maximum masticatory forces for molars, and almost twice for premolars (>1000 N). The results of the present study were similar to those obtained by Hassouneh et al. [32] in the group of CAD/CAM resin based-materials. Despite the discrepancies in the type of design, our results are in accordance with the results obtained in a recent retrospective study that showed 100% tooth survival and 96.8% restoration survival after four years in endodontically treated teeth reconstructed with onlay composite restorations [47].

When compared to full-coverage crowns, different in vitro studies have shown that endocrowns had a higher fracture resistance [27,41,42]. Clinical evidence from a meta-analysis showed a success rate for endocrowns on molars and premolars between 94% and 100% in studies with 36 months follow-up [48]. However, in a recent systematic review, it has been observed that, when only premolars were analyzed, survival decreased to 68–75% [45]. Therefore, it would be interesting to know if preparation design plays a role on fracture resistance. In this sense, Samran et al. [40], compared fracture resistance at different locations of 2 mm of ferrule (circumferential ferrule, buccal ferrule, lingual ferrule, buccal and lingual ferrule and teeth without ferrule) in endodontically treated mandibular premolars with fiber posts and crowns. The results showed no statistically significant differences between groups except between the control group (natural teeth with no posts, no crown) and groups with buccal and lingual 2 mm-ferrule and no ferrule. These authors concluded that the location of the ferrule had no significant effect on the fracture resistance of endodontically treated mandibular premolars. In the present investigation, we observed that the preparation design and the depth of the interproximal cavities might have an influence on fracture resistance. Thus, when the distance from the internal and interproximal margins to the bone crest reduced, fracture resistance of the restoration increased. This could be explained due to the increase in the volume of the restoration and its greater retention. However, when the margins of the restoration were located at the level of the simulated alveolar ridge, fracture resistance was drastically reduced (780.67 N). This suggests that a minimal supracrestal healthy dental structure favors fracture resistance.

As in other in vitro studies, it is difficult to extrapolate the results of this study directly to a clinical situation since masticatory forces were simulated only at a certain angle. A static loading model was used, without considering the behavior of these restorations during dynamic tests and temperature changes. Furthermore, since this is an in vitro model, the periodontal ligament is not present and, of course, neither is its influence. It should not be forgotten, that these results do not allow evaluating what the soft tissue response would be at the different heights of the restoration margins. Results in the literature have shown that the placement of the subgingival margins, lead to an inflammatory response in the support tissues, with an increase in bleeding, gingival index, loss of attachment and changes in the subgingival microbiota [49]. Future research should evaluate the design of these cavities under dynamic loads, thermal-cycling, and different materials to determine whether CAD/CAM resin materials are best suited for this cavity design as well as the biologic response.

## 5. Conclusions

Within the limitations of this in vitro study, the location of the interproximal margins in overlay endocrown restorations seems to influence the fracture resistance. Thus, preparations with margins located 0.5 mm and 1 mm from the alveolar crest showed significantly higher fracture resistance than margins located at 2 mm distance from the alveolar crest. From a clinical point of view, the analysis of the obtained results seems to demonstrate that the approach when performing crown lengthening or orthodontic extrusions, might be more conservative, being only necessary to preserve the anatomical distance for the correct conformation of the biological width. Also, the resistance conferred by this type of restoration justifies a less invasive preparation than what was regularly needed with the use of crowns.

Since this was an in vitro study, further clinical studies should be carried out in order to evaluate the soft tissue response to the different apico-coronal positions of the restoration margins.

## Figures and Tables

**Table 1:** Main fracture resistance in the different treatment groups.

Group	Sample	Average (N)	SD	Minimum (N)	Maximum (N)	Range (N)
Group A (Control)	10	859.61	267.951	515.4	1280.0	764.6
Group B	10	1053.9	333.985	628.0	1418.0	790.0
Group C	10	1124.6	291.172	525.0	1420.0	895.0
Group D	10	780.67	183.269	535.0	1077.0	542.0
Total	40	954.695	299.391	515.4	1420.0	904.6

N: Newtons; SD: Standard Deviation.

## Data Availability

The data presented in this study are available on request from the corresponding author.
